# Simulation-Based Enhancement of Patient Safety During Intrahospital Transport of Trauma Patients With COVID-19: A Helipad Scenario

**DOI:** 10.7759/cureus.67484

**Published:** 2024-08-22

**Authors:** Banu Arslan, Mehmet Necmeddin Sutasir, Mucahit Kapci, Semih Korkut, Selim Altinarik

**Affiliations:** 1 Emergency Medicine, Basaksehir Cam and Sakura City Hospital, Istanbul, TUR; 2 Emergency Health Services, Istanbul Provincial Health Directorate, Istanbul, TUR

**Keywords:** helipad, patient transport, simulation, pandemic, patient safety

## Abstract

Trauma resulting from accidents, violence, or war claims over five million lives annually, with traffic accidents and falls being predominant causes. The COVID-19 pandemic has posed unprecedented challenges in trauma care. Even though the number of injuries decreased during the lockdown period, the transportation of trauma patients became even more challenging due to concerns about infection control and the need for enhanced protective measures. This simulation workshop was conducted in a controlled environment to test and refine protocols for the safe transport of trauma patients with COVID-19. Our goal was to develop comprehensive guidance on the intrahospital transportation of these patients, ensuring the highest level of patient care and safety. We detail a five-step approach from preparation to the pre-landing, initial assessment, patient transfer, and patient handover phases, emphasizing adherence to protocols, equipment readiness, and personal protective equipment (PPE) use. The primary issues we encountered were related to time management, the decision-making process for applying lifesaving procedures in an open environment, and the risk of cross-infection. The workshop underscored the importance of swift and coordinated care, balancing life-saving interventions with efficient transport to a definitive care facility.

## Introduction

More than five million people die annually due to injuries resulting from road traffic crashes, falls, drownings, burns, poisoning, violence, or acts of war [1]. Among these, traffic accidents are the most reported mechanism of trauma, while falling and work accidents take second place [2]. Several studies have reported a significant reduction in the number of traumas during the COVID-19 pandemic lockdown period [3-5]. However, the pandemic has brought unprecedented challenges to every step of trauma patient care, from prehospital care to definitive care.

The importance of treating trauma patients in the right place at the right time to ensure safe and high-quality care is indisputable. Time is a critical factor in trauma care, as swift intervention can significantly enhance the chances of survival and reduce long-term complications. Several studies have indicated that treating severely injured patients in Level 1 trauma centers is associated with improved outcomes [6,7]. Trauma centers are equipped to handle severe cases, providing immediate and specialized care, including surgery and critical care services. Although Level 1 trauma centers are ideal for many severely injured patients, immediate transfer may not always be feasible or necessary. Thus, some patients may require secondary transport to a higher level of care [8]. Patient transport, whether from one hospital to another or from one physical location to another within the hospital, is a high-risk process. Moving patients who are already traumatized to an environment with changed care settings is particularly challenging. Various adverse events, including the deterioration of vital parameters, malfunctioning of equipment, and displacement of tubes and lines, may occur during the transportation process [9]. Additionally, factors such as time delays, a low level of staff training, limited access to essential equipment, and poor communication between role players can influence morbidity and mortality [10]. From this perspective, early recognition of life-threatening symptoms, delivery of pre-hospital medical interventions, and safe transport to a definitive care facility are vital.

The COVID-19 pandemic highlighted concerns faced by health providers. The novel COVID-19 is highly contagious, and transmission can occur under the condition of prolonged exposure to high concentrations of aerosols in a relatively closed environment [11]. Healthcare workers are at a high risk of infection during intubation due to close contact with infected patients and contaminated materials [12]. Additionally, the risk of disease transmission appears to be higher in emergency medical services (EMS) providers compared to in-hospital healthcare workers due to limited space, time, and patient information [13].

The COVID-19 pandemic led us to reassess our intrahospital patient transport process and implement additional measures to ensure the safety of the patient and transportation team. Hereby, we present our simulation workshop, sharing our experience in the intrahospital transportation of a trauma patient infected with COVID-19. Through this workshop, we demonstrated every important stage of patient transfer from the helipad to the emergency department (ED), ensuring the safest and highest level of patient care provided. Our objective was to develop comprehensive guidance, highlighting the importance of systematic training, detailed checklists, and strict adherence to protocols to minimize infection risks. We hope that our protocol will be a helpful resource for others in ensuring the safe transportation of trauma patients infected with COVID-19.

## Technical report

5-steps of intrahospital patient transfer technique

**Figure 1:**
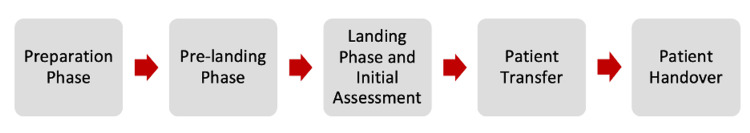
Phases of patient transfer

Here is the stages of patient transfer from the helipad to the ED (Figure 1). 

Preparation Phase 

On February 8, 2021, at 10:55 am, the National Air EMS informed our hospital’s EMS coordinator about a 52-year-old male patient. The patient suffered serious head and leg injuries from an earthquake in rural Istanbul and was scheduled for air ambulance transfer to our emergency department. The patient reported being conscious; however, he exhibited a lack of cooperation and orientation. The Glasgow Coma Scale was 14, S1 (+) and S2 (+). His heart rate was 120/min, body temperature was 35.6°C, oxygen saturation was 96%, systolic blood pressure (SBP) was 122 mm/hg, and diastolic blood pressure (DBP) was 68 mm/hg. The COVID-19 status was not known. Estimated arrival time was stated as 11:20 am. 

At 10:58 am, the hospital’s EMS coordinator activated the intrahospital patient transportation team, and the preparation phase had commenced. The team members were previously assigned, and their lists were regularly communicated to the EMS coordinator on a monthly basis. The operation was supervised by an emergency medicine (EM) physician and supported by a highly skilled team. In addition to the EM physician, the team comprised four nurses and a substantial group of security, transport, and technical staff. The entire team received training to prevent potential work-related injuries, including rotor blade injuries, chemical exposure, and back injuries, prior to the start of the simulation. 

At 11:04 am, the security staff initiated the clearance of the route from the helipad to the patient room in the ED. Concurrently, the medical team started to conduct a meticulous review of the equipment checklist (Table 1) to confirm the presence of all listed items in the transportation bag. They ensured comprehensive preparedness to intervene in any adverse events that could potentially affect the patient’s clinical status. Personal protective equipment (PPE) and other materials essential for emergency procedures were prepared and verified. Given the elevated number of COVID-19 cases at the hospital, the team was already equipped with their PPE. 

**Table 1:** Equipment checklist IV: intravenous; IO: intraosseous

Systems	Equipment	Functions and Essentials
Airway	Bag valve masks	Masks of various sizes
	Oropharyngeal and nasopharyngeal airways	Airways of various sizes
	Laryngoscope or video laryngoscope	Blades of alternate design and sizes
	Endotracheal tubes	Tubes of different sizes
	Supraglottic airway	Laryngeal mask airway w lubricant gel
	Retroglottic airway	Laryngeal tube, combi tube, king tube
	Emergency cricothyrotomy kit	Syringe, neck strap, and connecting tube
	Portable suction	Move secretions from the respiratory tract
	Cervical collar	Appropriately sized collar
	Waveform capnography	Monitors end-tidal CO2 levels to assess ventilation adequacy
Breathing	Chest tube insertion kit	Pleural drainage system, chest tubes of various sizes, scalpel blades in different sizes, angled clamps, needle driver, scissors, curved sutures of size 1.0 or larger, 10-ml and 20-ml syringes
Circulation	Portable monitor with an illuminated display	Record heart rhythm and vital parameters
	Defibrillator with a fully charged battery	Treat life-threatening arrhythmias
	Pad of sterile cloth and gauze	Compress in case of any bleeding
	Emergency splints	Provide neurovascular protection
	Tourniquet	Control life-threatening hemorrhage
	Intravenous fluids	Prevent or treat dehydration (NS, 0.9NaCL)
	Drugs: vasopressors, analgesics, resuscitation drugs, and the drugs used for rapid sequence intubation	Vasopressors, atropin, calcium, magnesium, amiodarone, lidocaine, ketamine, fentanyl, etomidate, tranexamic acid, etc.
	Various syringes and other vascular access devices	Peripheral IV lines, central catheters, IO access device
Other	Warming blanket, nasogastric tube, and additional batteries	

Pre-landing Phase

**Figure 2:**
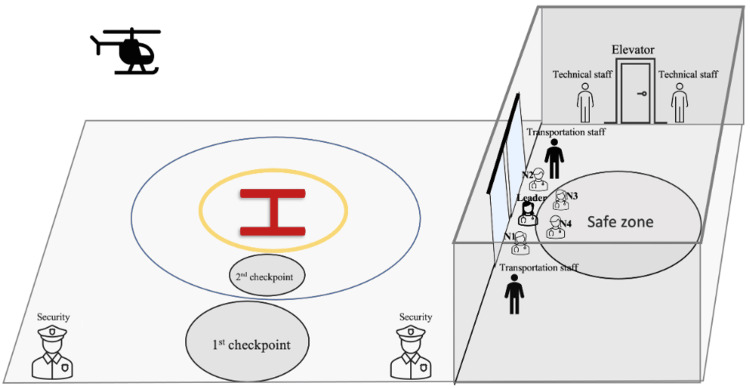
Pre-landing Phase: security team establishing ground safety around the helipad N1: nurse number 1; N2: nurse number 2; N3: nurse number 3; N4: nurse number 4 Image credits: Author Banu Arslan

At 11:16 am, the team positioned themselves in the designated safe zone, awaiting the arrival of the helicopter at the hospital (Figure 2). Ground safety measures in and around the helipad area were established by the security team. Once the security team confirmed ground safety using mobile communication tools, landing permission was granted to the helicopter. Concurrently, technical personnel ensured that the runway doors and elevators were designated for use exclusively by the transportation team from that point onward. 

Landing Phase and Initial Assessment

**Figure 3:**
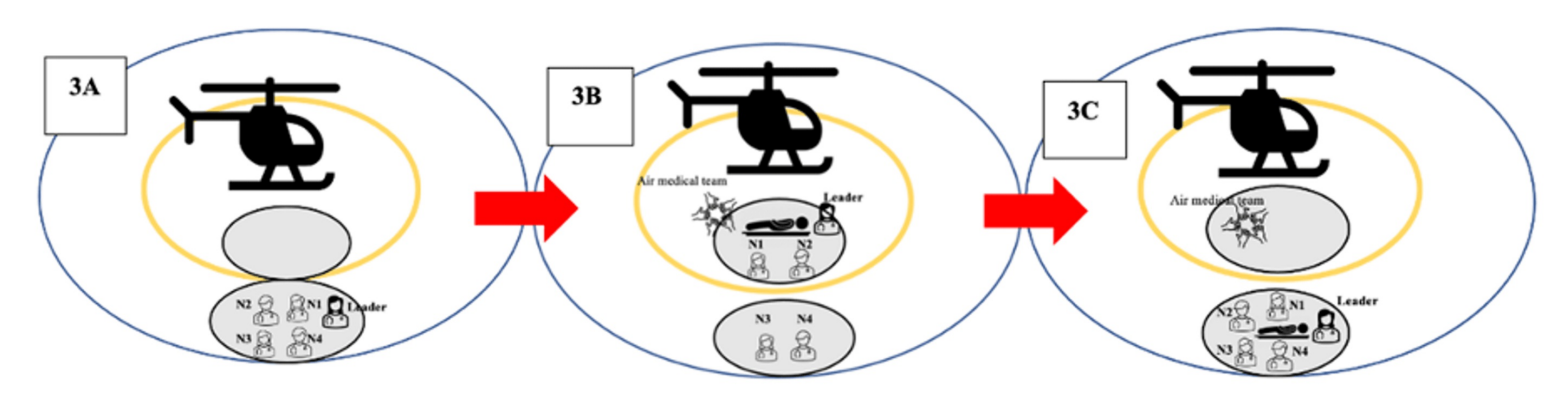
Patient transportation process 3A: the transportation team proceeds to the first control point; 3B: patient handover between air medical crew and transportation team; 3C: initial assessment at the first checkpoint N1: nurse number 1; N2: nurse number 2; N3: nurse number 3; N4: nurse number 4 Image credits: Author Banu Arslan

**Figure 4:**
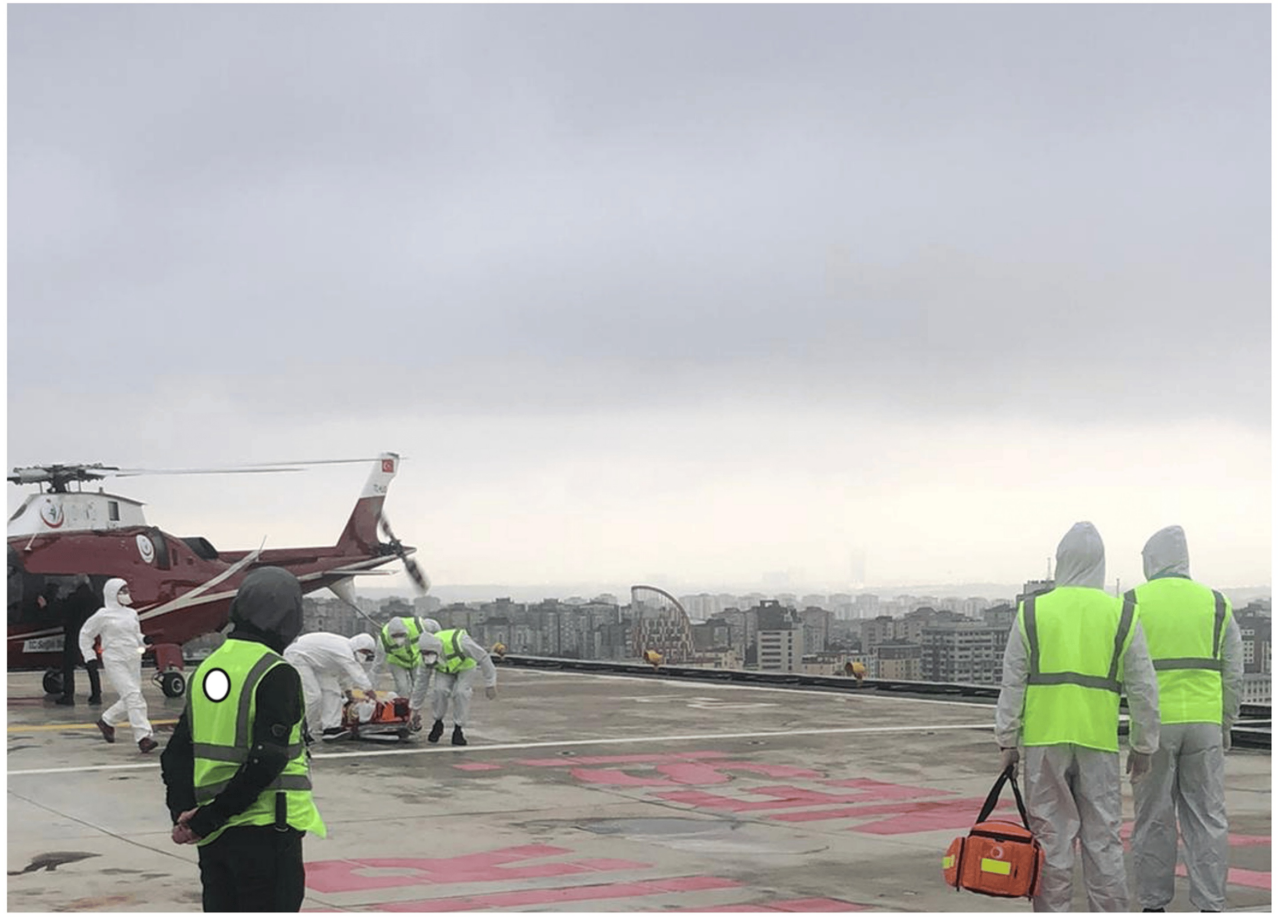
Patient handover between air medical crew and transportation team (also shown in Figure 3B) The team is receiving the patient at the seconnd check point

Upon landing at 11:19 am and with the rotors coming to a complete stop, security clearance was signaled on the helipad. A hand signal allowed the transportation team to proceed to the first checkpoint (Figure 3A). Then, the EM physician and two nurses approached the second checkpoint, placing the patient on a stretcher in coordination with the air medical team (Figure 3B, Figure 4). The patient’s identity and history were verbally confirmed here. Subsequently, the team promptly returned to the first checkpoint (Figure 3C).

At the first checkpoint, the team consisted of the EM physician and four nurses. Among the four nurses, two were fully equipped with all the drugs and instruments necessary for airway management, oxygenation, ventilation, hemodynamic stabilization, and resuscitation. The remaining two nurses were tasked with closely monitoring the patient. 

At the first check point, the patient underwent an initial assessment in accordance with the Advanced Trauma Life Support (ATLS) guidelines. The patient appeared restless and showed signs of distress. Although conscious, he displayed non-cooperative behavior, possibly due to the head trauma and intense leg pain. The airway was patent with no signs of respiratory distress. Both lungs were inflating equally, with clear lung sounds. Comprehensive patient monitoring, which included continuous electrocardiogram (ECG) monitoring, non-invasive blood pressure, pulse oximeter, and temperature, was initiated. The patient demonstrated sinus tachycardia, and his oxygen saturation was measured at 95%. Blood glucose was 144 mg/dl. The left leg exhibited a deformity with no active bleeding. Treatment with IV saline infusion and supplemental oxygen was initiated. 

It is important to note that if the patient had required any lifesaving procedures, such as cardiopulmonary resuscitation, defibrillation, active bleeding control, neck collar application, adrenaline administration, and shock treatments, these should have been performed at the first checkpoint.

Patient Transfer 

At 11:24 am, the team completed the initial assessment and proceeded to the designated safe zone. In the safe zone, the team reassessed the patient and ran through the departure checklist shown in Table 2. The EM physician reassessed the patient for airway stability, adequate perfusion, and adequate ventilation. Patient was hemodynamically stable except for sinus tachycardia. The GCS was 14/15 (eye opening: 4, verbal response: 4, motor response: 6). 

**Table 2:** Departure checklist AVPU: alert, verbal, pain, unresponsive

ABCDE Assessment	Critical Aspects of Patient Care	Equipment	Other Considerations
Airway	Safe? Stable? Secured? Need for intubation?	Peripheral IV devices	Adequate access? Drugs, pumps, and lines are secured?
Breathing	Adequate ventilation? Oxygen saturation >95%? Need for chest tube? Chest drain is effective?	Drugs	Enough supply? Intravenous fluids, vasopressors, analgesics, muscle relaxants, sedatives, and resuscitation drugs
Circulation	Adequate perfusion? Need for vasopressor? Need for IV fluids? Hemorrhage controlled? Splint? fasciotomy?	Monitoring	Electrocardiography, pulse oximetry, blood pressure, end-tidal carbon dioxide, and temperature monitors are attached and working?
Disability	Glasgow Coma Scale? AVPU? Blood glucose? Pupillary light reflexes? Lateralization?	Essentials in trauma care	Cervical collar? Trauma board? Pelvic binder? Suturing, thoracotomy, chest tube, and fasciotomy kits?
Exposure	Risk for hypothermia? Need for a warm blanket?	Other	Enough oxygen supply? Battery?

There was a large swelling accompanied by a 4 cm laceration on the left parietal area of the patient’s head. Pupils were equal and reactive to light. His neck showed no tenderness or deformity. No tenderness or signs of injuries were observed in the chest, abdomen, or back. The EM physician prescribed IV analgesic and applied longitudinal traction, subsequently splinting the left leg. Due to the increased risk of hypothermia, the patient was covered with a blizzard blanket. Simultaneously, the nurses conducted a thorough check of patient monitoring, peripheral IV accesses, and other equipment to ensure proper functioning before departure. 

**Figure 5:**
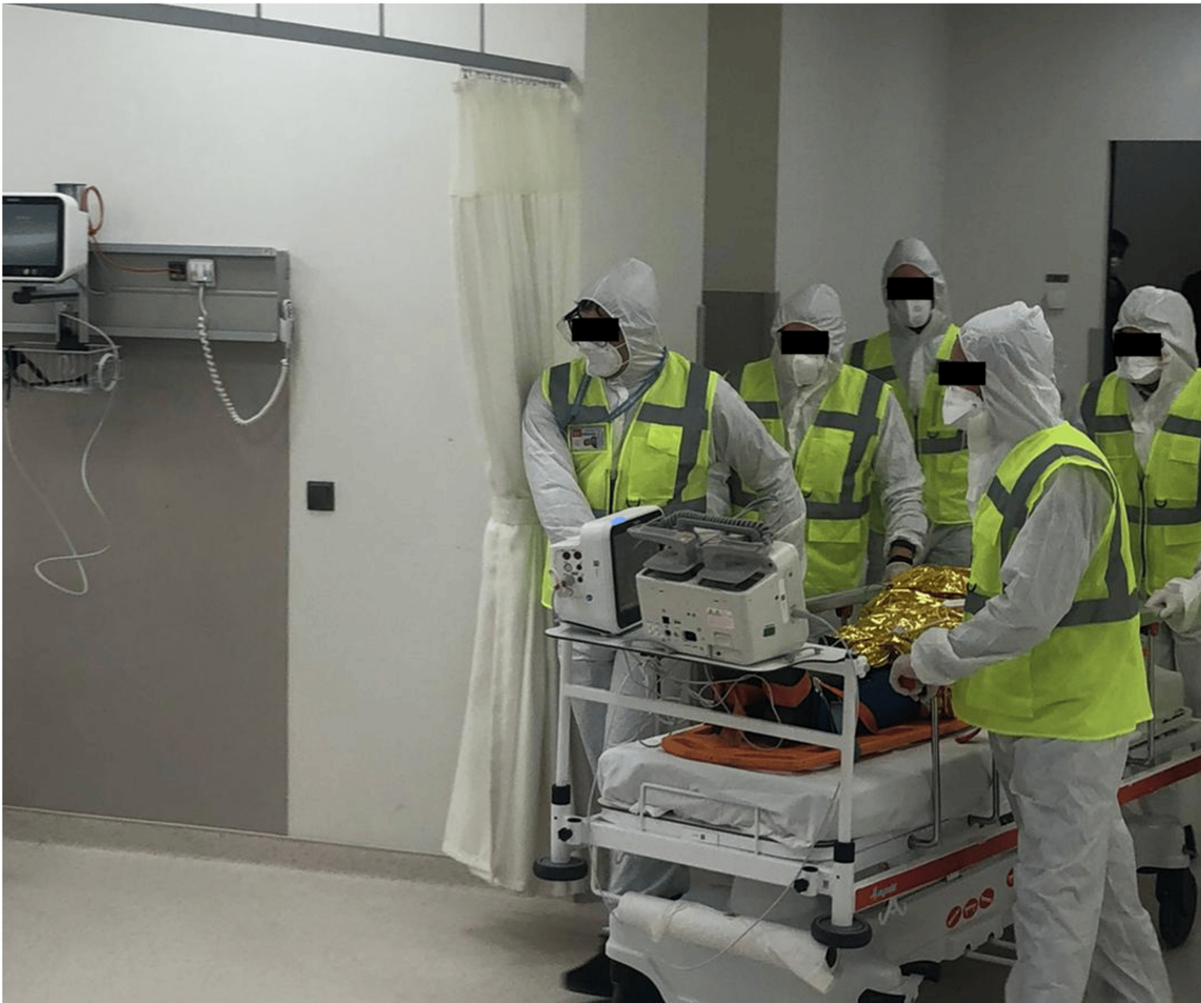
Transportation to the emergency department (also shown in Figure 3E)

At 11:31 am, the technical staff opened the elevator doors, and the patient’s transportation to the emergency department started (Figure 5). Throughout the transportation, the patient was closely monitored, and detailed documentation of clinical progress at each stage was recorded.

Patient Handover 

**Figure 6:**
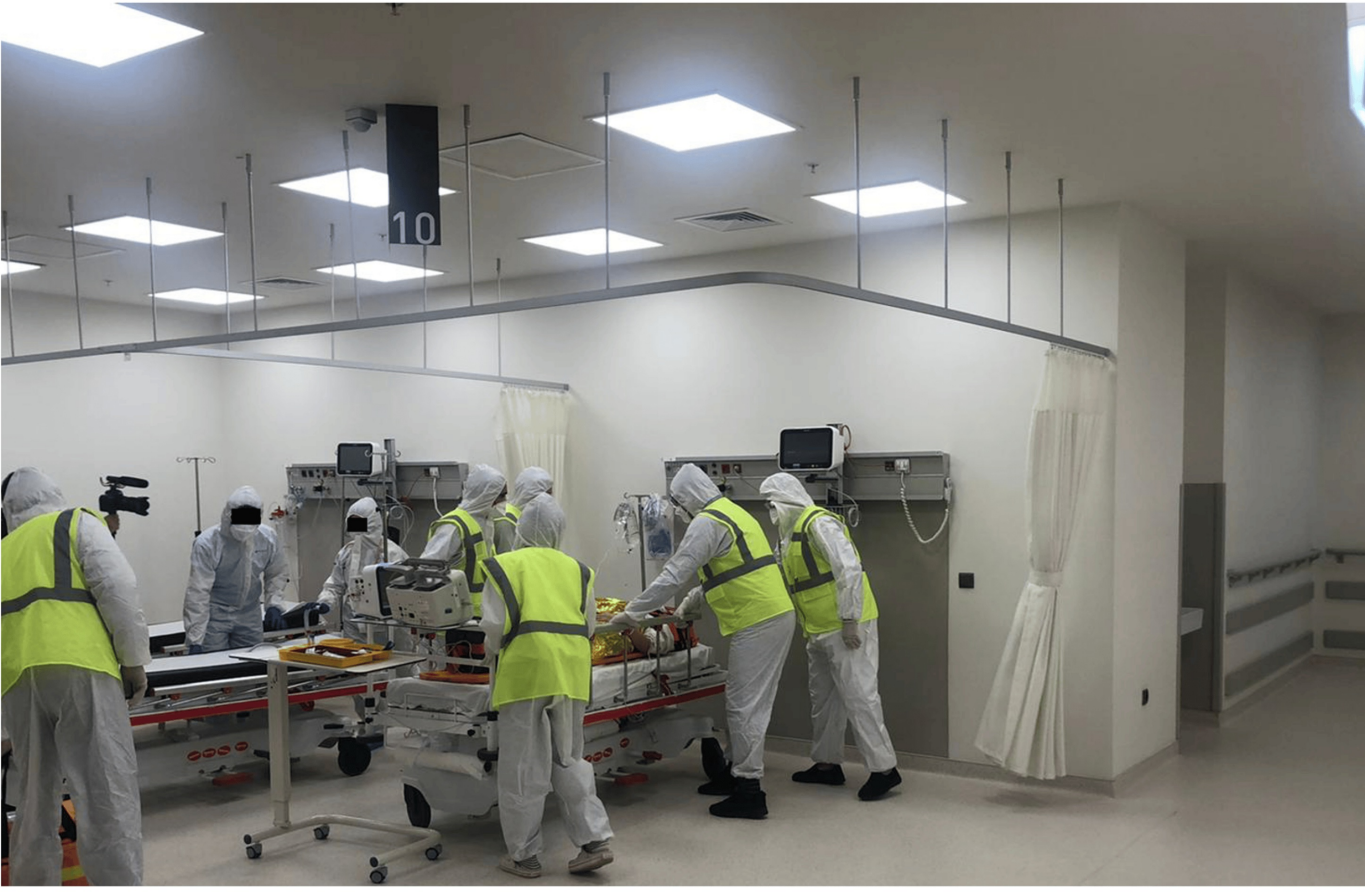
Patient handover (also shown in Figure 3F)

At 11:35 am, the team reached the ED, and the patient was handed over to the trauma team. A comprehensive patient transfer form, encompassing all stages of the transfer, was communicated to the trauma team. In addition to basic information such as the patient's name, age, and reason to transfer, the patient handover document included details on clinical progress involving the status of vital signs, clinical events, and treatments administered before and during the transfer. Furthermore, the patient forms prepared by the air medical crew were incorporated into our transfer documents (Figure 6). 

## Discussion

This simulation workshop detailed a comprehensive and well-coordinated approach to intrahospital patient transport, with a particular focus on the safety of trauma patients infected with COVID-19, as well as the safety of the transportation team. The safety of patient transport hinges on several factors, including staff training, adherence to established hospital protocols, the use of checklists, the availability of necessary equipment, efficient time management, and effective communication between role players [14]. Systematic training for team members showcases the importance of proactive measures in responding to critical cases. In this simulation, our team adhered to our established patient transportation protocols, utilized checklists, and exhibited well-orchestrated communication. 

Survival following severe trauma is not solely dependent on short rescue times but on the effective utilization of this time. Health professionals must balance the application of necessary procedures with minimizing pre-hospital time. It is highly recommended that only lifesaving procedures should be administered at the scene [15,16]. During our initial assessment phase, the team employed the ATLS approach, intervened to the hemodynamic compromise, and immediately proceeded to the safe zone as recommended. Critically ill patients are prone to instability during transfer [17]. The primary objective of treatments should be to optimize oxygen transport, monitor hemodynamics, manage bleeding, and control hypothermia [18]. Our team completed a systematic reassessment and completed the departure checklist at the safe zone, ensuring all necessary interventions were completed before proceeding to the ED. In our case, the patient had a suspected femur fracture; therefore, traction and splinting were applied promptly to alleviate pain, prevent bone protruding through the skin, and control bleeding. Additionally, the patient was covered with a blizzard blanket to mitigate the risk of hypothermia. 

The management of trauma patients has become increasingly complex amid the pandemic due to the COVID-19 virus's highly transmissible nature. Healthcare workers, particularly EMS providers, face heightened cross-infection risks [12,13]. Reverse-transcriptase polymerase chain reaction (RT-PCR) remains the gold standard for COVID-19 diagnosis, yet limited resources and time constraints often hinder healthcare workers from promptly reaching a conclusive diagnosis on-site. Given these challenges, we advocate a precautionary approach, treating every patient as potentially positive for the virus. In our simulation, the patient arrived with an undisclosed COVID-19 status. Given the surge in COVID-19 cases in our area, the team adhered strictly to the standardized PPE protocols throughout the entire transportation process.

Learnings from this simulation

Our simulation revealed several important insights regarding our patient transfer process, each presenting unique challenges. The primary issues we encountered were related to time management, the decision-making process for applying lifesaving procedures in an open environment, and the risk of cross-infection. Below, we discuss each challenge in detail, starting with time management.

Time Management

In this simulation, the patient transfer from the helipad to the ED lasted approximately 16 minutes. Initially, our goal was to limit both the initial assessment and the patient assessment in the safe zone to five minutes. However, we spent seven minutes in the safe zone. We acknowledged that the duration could vary depending on the patient's clinical condition. In addition, the process of moving the patient from the safe zone to the ED took four minutes, exceeding our planned timeframe by two minutes. The delay was primarily attributed to the time-consuming task of fitting the nine-person team, the patient on the stretcher, and the medical equipment (including a portable oxygen tank) into the elevator. For future simulations, we are contemplating the option of excluding security staff to streamline the process before taking the elevator.

Decision-Making for Lifesaving Procedures in an Open Environment

Adverse weather conditions, particularly during wintertime, can present substantial challenges. Frequent freezing temperatures and windy days necessitate meticulous attention to patient safety. To ensure the well-being of patients, we recommend relocating the initial assessment from the first checkpoint to the safe zone situated on the same floor as the helipad but inside the building. This modification will create a controlled environment for conducting the initial assessment and applying life-saving procedures, particularly during unfavorable weather conditions. 

Risk of Cross-Infection

While in the elevator, the team encountered a heightened risk of COVID-19 transmission. Despite our team's strict adherence to cross-infection prevention protocols, the potential for close contact with infected patients and contaminated materials remained a significant concern. For future simulations, we recommend deliberating on additional safety measures such as minimizing the number of staff in elevators, reviewing and upgrading PPEs, having regular staff training sessions, or improving ventilation in elevators.

## Conclusions

This simulation workshop underscored the complexities of intrahospital transport of trauma patients, especially in the context of COVID-19. Key issues such as time management, decision-making in adverse conditions, and cross-infection risks were highlighted. This workshop revealed the need for more efficient coordination and revising team composition and procedures to prevent delays. Relocating the initial assessment to a more controlled environment and exploring additional safety measures for cross-infection became evident. While timely and accurate execution of lifesaving procedures remains paramount, the transport team must balance these actions with the need for rapid, safe patient transfer to areas equipped for advanced care. Future research should focus on evaluating the impact of these proposed changes on patient outcomes and exploring other potential improvements in the transport process.
